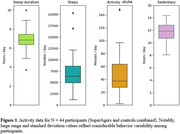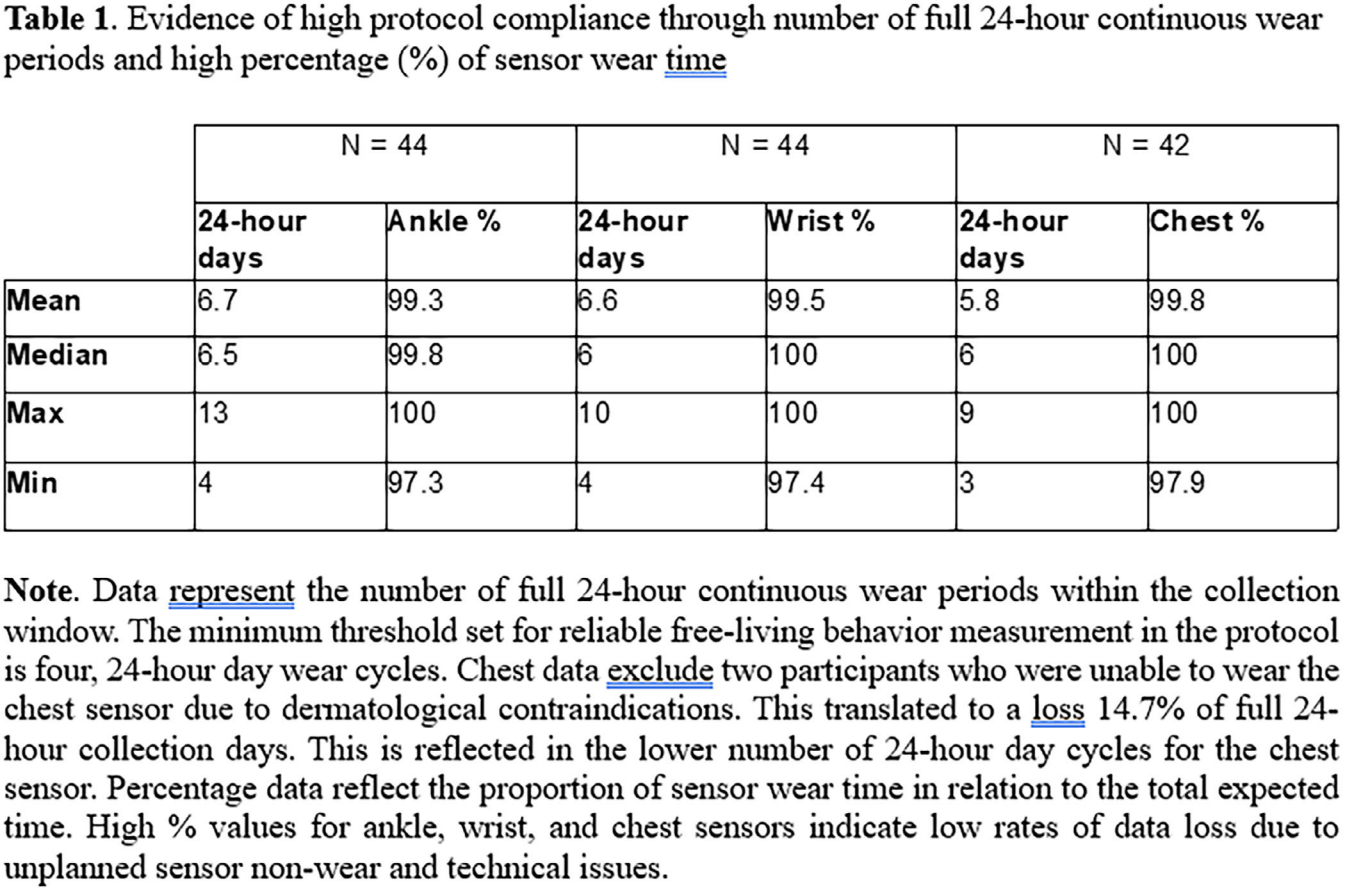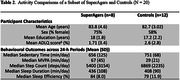# Health‐Related Behaviours in Octogenarians and Nonagenarians with Robust Cognitive Longevity: Progress and Initial Data from thee SuperAging Research Initiative

**DOI:** 10.1002/alz.089381

**Published:** 2025-01-09

**Authors:** Angela C Roberts, Karen Van Ooteghem, Bill McIlroy, Ivan Culum, Kit B Beyer, Vanessa Thai, Nimrit Aulakh, John Li, Ciara Dunne, Andrew Lim, Richard H Swartz, Elizabeth Finger, Amanda Cook Maher, Ozioma C Okonkwo, Felicia Goldstein, Matthew J. Huentelman, Changiz Geula, Emily J Rogalski

**Affiliations:** ^1^ Canadian Centre for Activity and Aging, London, ON Canada; ^2^ Northwestern University, Evanston, IL USA; ^3^ Western University, London, ON Canada; ^4^ University of Waterloo, Waterloo, ON Canada; ^5^ Toronto Rehabilitation Insitute, Toronto, ON Canada; ^6^ Sunnybrook Health Sciences Centre, Toronto, ON Canada; ^7^ Temerty Faculty of Medicine, University of Toronto, Toronto, ON Canada; ^8^ University of Western Ontario, London, ON Canada; ^9^ University of Michigan, Ann Arbor, MI USA; ^10^ University of Wisconsin, Madison, WI USA; ^11^ Emory University School of Medicine, Atlanta, GA USA; ^12^ The Translational Genomics Research Institute (TGen‐ an Affiliate of City of Hope), Phoenix, AZ USA; ^13^ Northwestern University Feinberg School of Medicine, Chicago, IL USA; ^14^ University of Chicago, Chicago, IL USA

## Abstract

**Background:**

SuperAgers—individuals age 80+ with episodic memory performance at least as good as those 20‐30 years younger—provide a unique perspective on cognitive resilience and resistance in aging. The SuperAging Research Initiative (SRI), spearheaded by The University of Chicago and involving multiple academic partners, investigates factors underpinning robust cognitive aging. One key SRI project, leverages a fully remote data collection paradigm to: 1) discern activity patterns that characterize SuperAgers and 2) explore the 'complexity hypothesis in aging'—whether dynamic physiological responsiveness is a hallmark of exceptional cognitive aging. Here we report on feasibility and initial outcomes from this project.

**Method:**

Participants don wearable sensors, including an ECG sensor (chest) and two inertial measurement units (wrist, ankle), for a 10–12‐day period of continuous data collection whilst performing their usual daily activities. The protocol includes a virtual orientation and periodic check‐ins to ensure wear‐compliance and provide technical support. Structured sensor‐wear breaks facilitate protocol compliance and acceptance.

**Result:**

To date, recruitment efforts have led to enrollment of 91 individuals (Mean age = 84.1 years; 62 women), which approximates half of the total SRI sample. Reasons for ineligibility or non‐enrollment include dermatological contraindications, ‘busy’ lifestyles, and perceived participation burden. Initial analyses of 44/58 participants who have completed data collection show exceptional adherence (>95% sensor wear compliance) (Table 1). Limited data loss due to sensor non‐wear and/or sensor failure demonstrates high data quality (Table 1). Notably, participants demonstrate a high level of independence, with study partner assistance required in < 5% of cases. Withdrawals have been minimal (3%) and primarily attributed to skin irritation due to undisclosed dermatological contraindications. Initial data are provided in Figure 1 and Table 2 including daily activity summary data and group comparisons between SuperAgers and Controls. Updated data will be presented at the conference.

**Conclusion:**

Wearable technologies are feasible for remotely assessing daily activities of octogenarians and nonagenarians with high compliance. Objective quantitative data hold promise for expanding our understanding of lifestyle factors relevant to superior cognitive aging. Insights from this study may influence preventive intervention strategies against age‐associated neurological diseases and in support of enhancing cognitive longevity.